# Supplementary material for: Striato-pallidal oscillatory connectivity correlates with symptom severity in dystonia patients
Source: Nat Commun. 2024 Oct 1;15:8475. doi: 10.1038/s41467-024-52814-4 (PMC11442513; doi:10.1038/s41467-024-52814-4)
Supplement: Supplementary file 1 — Supplementary Information [file 41467_2024_52814_MOESM1_ESM.pdf]

Supplementary Materials

Surgical procedure

All patients underwent bilateral DBS in the globus pallidus internus (GPi) at Charité University Hospital, Berlin (n=7) or Medizinische Hochschule Hannover (n=3). Electrodes were targeted at the posteroventrolateral portion of the GPi. Permanent octopolar DBS leads (Boston Scientific Vercise) were implanted under general anaesthesia in all patients. The stereotactic target points were determined by a combined approach of indirect standard coordinates followed by direct refinement according to individual anatomy of nuclei as assessed by structural preoperative MRI (see below). The preliminary target for GPi (reflecting the center of the most distal electrode contact) was identified 2-3 mm anterior, 19-22 mm lateral and 4 mm below the midcommissural point. Under these circumstances, with a trajectory that is angled by 70–80° in the sagittal plane and by 75–85° in the coronal plane, the more rostral contact pairs are likely to lie in the globus pallidus externus (GPe) and Putamen. Intraoperatively, the final electrode position was verified using microelectrode recordings and macrostimulation with tetanic stimulation to assess proximity to the internal capsule.

Preoperative MRI acquisition

In all patients, high-resolution T1w and T2w MRI scans were obtained preoperatively using a 3.0T clinical MRI scanner (Skyra or Vida Magnetom, Siemens, Erlangen, Germany). In addition, fast gray matter acquisition T1 inversion recovery (FGATIR) sequences were acquired in n = 7 patients. The complete MRI acquisition protocol consisted of a 3-plane localizing scout, a T1w 3-dimensional (3D) magnetization-prepared rapid acquisition gradient echo sequence, a T2w turbo spin echo (TSE) sequence, and a T1w 3D FGATIR sequence. A detailed overview of the acquisition parameters used for the protocol can be obtained from Supplementary table 3. Individual MRI imaging with FGATIR sequences allow a refined targeting to the transition between the middle and dorsal third of the GPi as depicted on axial FGATIR sequences.

Connectomic Analysis

The connectomic analysis involved a three-step process. Firstly, from the striatal recording contacts, volumes of 2 mm diameter[1] were projected as seed regions into an openly available group connectome based on fMRI images of 1000 healthy subjects from the Human Connectome Project[2]. This projection resulted in the creation of whole-brain functional connectivity maps for each patient. These maps were averaged across patients to visualize the

mean functional connectivity of all striatal recording sites. A similar approach has been used across several other publications.

1. Neumann, W.-J. *et al.* Functional segregation of basal ganglia pathways in Parkinson's disease. *Brain* **141**, 2655–2669 (2018).

2. Van Essen, D. C. *et al.* The Human Connectome Project: a data acquisition perspective. *Neuroimage* **62**, 2222–2231 (2012).

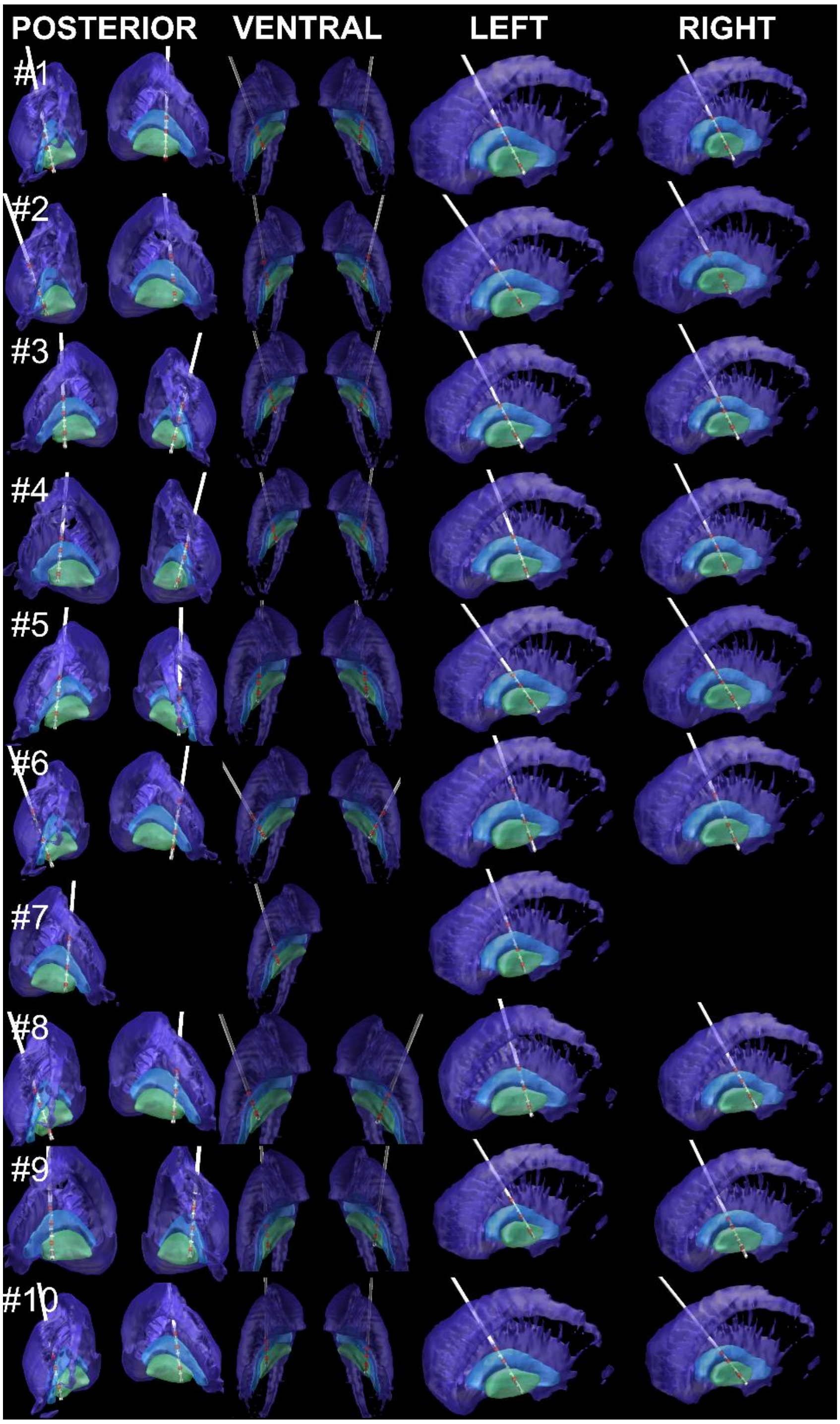

Supp. Figure 1: Different views of DBS-electrode localization with indication of recording sites for all subjects. Shown is the localization of DBS-electrodes within the basal ganglia nuclei according to the DISTAL atlas for subcortical structures of all included patients from the posterior view (column 1), dorso-ventral view (column 2) as well as the medio-lateral view of the left (column 3) and the right hemisphere (column 4) . The recording sites included in further analysis are labeled by red dots. For each patient, three recording sites per hemisphere are selected with one lying within the striatum (purple), one within the GPe (blue) and one within the GPi (green). Note that for patient #7 only the right hemisphere is shown, because the left DBS-electrode did not cover the striatum and was thus excluded from further analysis.

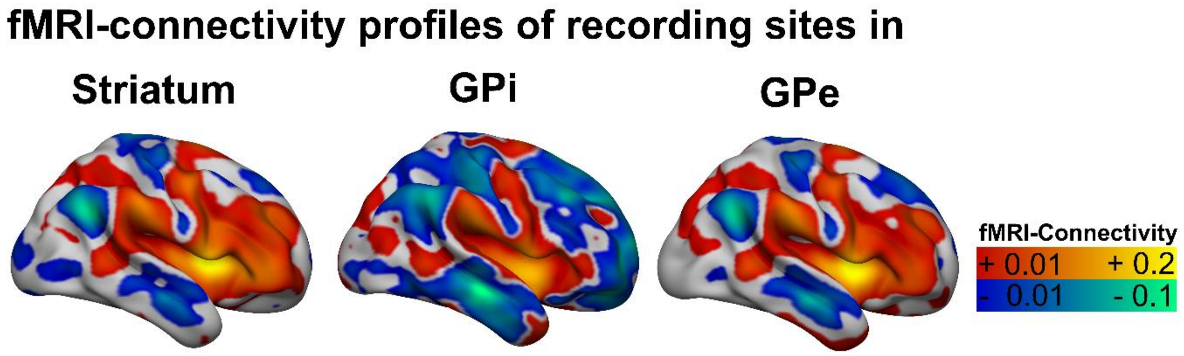

Supp. Fig. 2: Normative connectivity profiles of recording sites within the basal ganglia. The averaged functional connectivity of recording sites within each basal ganglia nucleus (left column: striatal recording sites; mid-column: GPi recording sites; right column: GPe recording sites) corresponds to the connectivity profile of their sensorimotor portion. For example, striatal recording sites show positive coupling to motor cortex, cerebellum and supplementary motor areas (yellow-red) and negative coupling to sensory cortices (blue-green). Note that these analyses have been performed within normative connectomes.

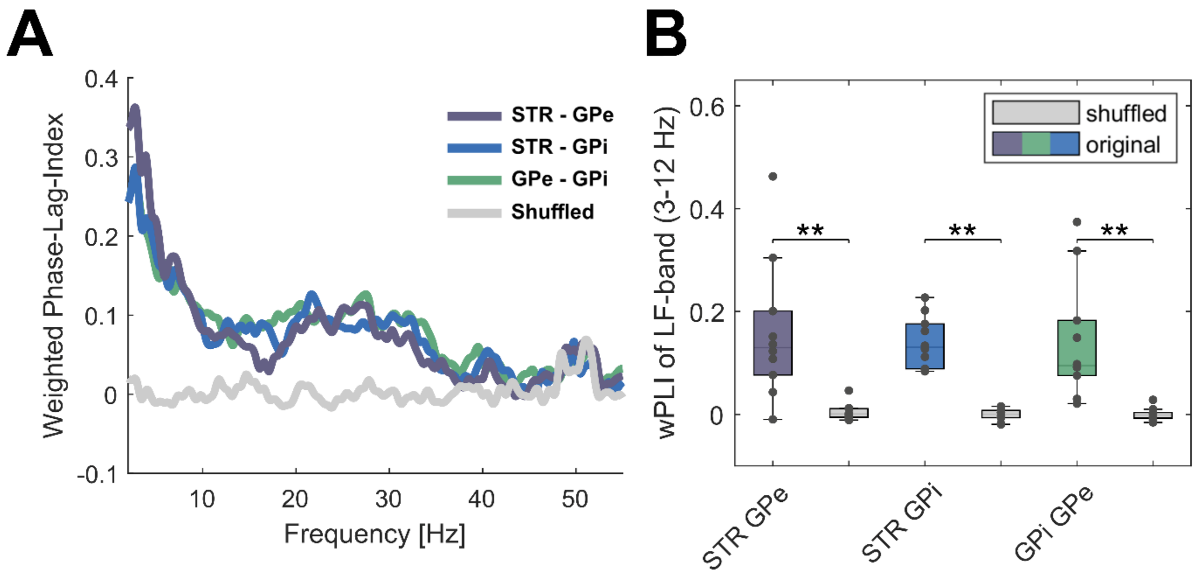

Supplementary Figure 3: Neuronal coupling across basal ganglia nuclei as measured by weighted phase-lag-index (wPLI). (A) Shown are averaged spectra of wPLI between striatum and GPe (purple), striatum and GPe (blue), GPe and GPi (green) and averaged shuffled wPLI (grey) across hemispheres. (B) All recorded basal ganglia structures are functionally coupled in the low-frequency band as shown by significantly higher wPLI values when compared to shuffled data. ** $p<0.01$. Black dots indicate mean value per hemisphere. In box plots, central marks indicate the median and edges the 25th and 75th percentiles of the distribution.

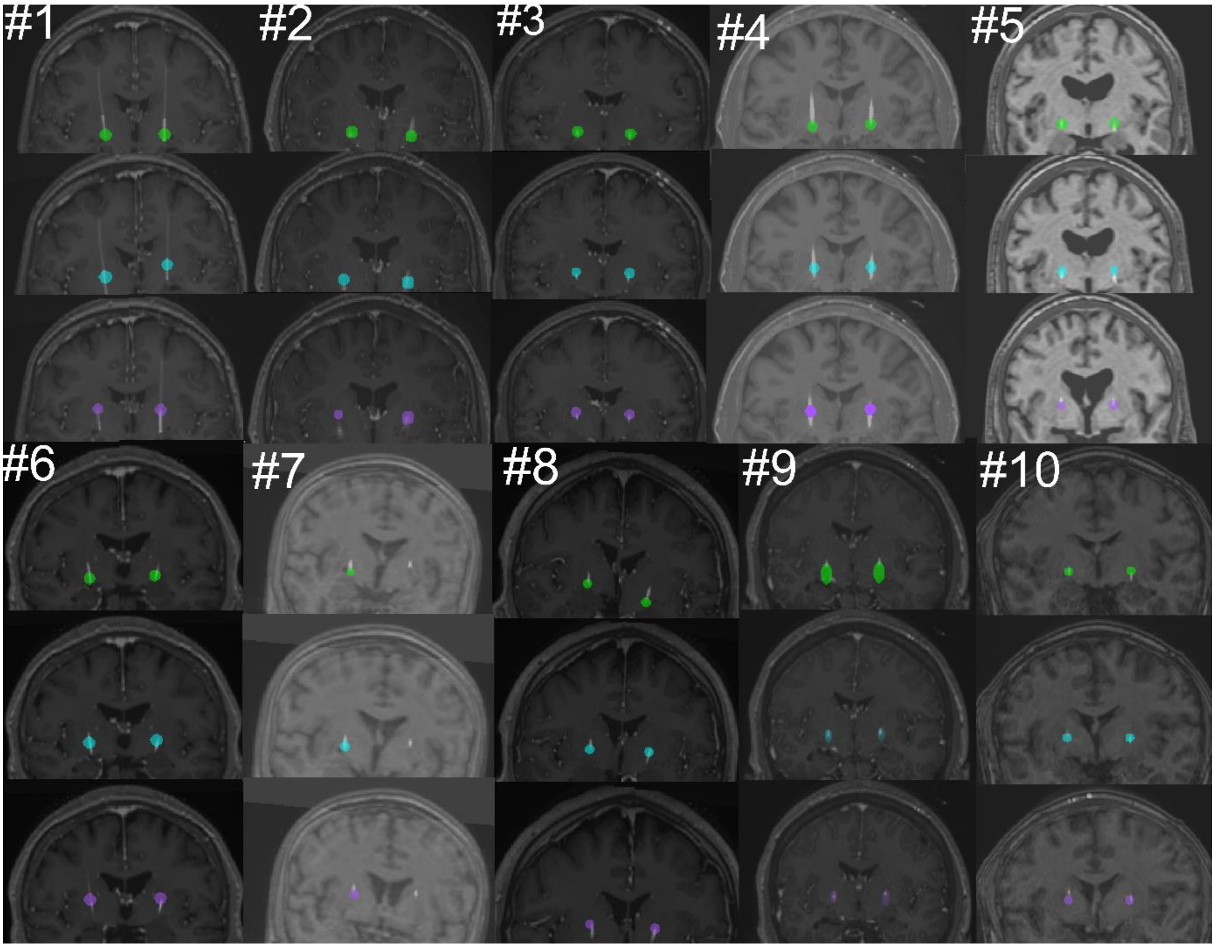

Supplementary figure 4. Overlay of preoperative MRI (T1) and postoperative CT with each contact-pair marked in native space. Shown are coronal planes in which the DBS-artifact and the recording site of the respective basal ganglia is visible (recording site is visualized with a radius of 4 mm in GPi=green, Gpe=blue, striatum=purple), separately for each patient. The size of the visualized recording site may vary according to the angle of the chosen MRI frame that best displays the electrode artefact. In subject #1, #6 and #8, the left and right DBS-electrode are not visible on the same coronal plane. In these cases, the coronal planes for each hemisphere have been merged for visualization purposes. Note that for patient #7 only the right hemisphere is shown, because the left DBS-electrode did not cover the striatum and was thus excluded from further analysis.

			Contact Pairs		
Subject	Case	Hem	STR	Gpi	Gpe
1	1	R	7-8	1-2	5-6
1	2	L	7-8	2-3	5-6
2	3	R	7-8	1-2	3-4
2	4	L	7-8	1-2	3-4
3	5	R	7-8	2-3	5-6
3	6	L	7-8	2-3	5-6
4	7	R	7-8	1-2	5-6
4	8	L	7-8	1-2	5-6
5	9	R	7-8	2-3	5-6
5	10	L	7-8	2-3	5-6
6	11	R	7-8	1-2	3-4
6	12	L	7-8	1-2	3-4
7	13	R	7-8	1-2	3-4
7	14	L	nan	nan	nan
8	15	R	7-8	1-2	4-5
8	16	L	7-8	2-3	5-6
9	17	R	7-8	1-2	4-5
9	18	L	7-8	2-3	5-6
10	19	R	7-8	2-3	5-6
10	20	L	7-8	2-3	5-6

Supplementary Table 1 : Contact pairs assigned to the striatum, GPi or GPe according to their localization using the DISTAL atlas. For visualization of individual electrode location, see Supp. Fig. 1.

BG nucleus	LF-Power			LF-Peak Frequency			Connectivity between BG nuclei	LF-iCOH		
	Cervical	Seg/Gen	P-Val	Cervical	Seg/Gen	P-Val		Cervical	Seg/Gen	P-Val
Striatum	5.8±1.8	5.8±1.8	0.9	8.1±2.1	9.0±3.3	0.4	STR-GPi	0.09±0.05	0.1±0.006	0.2
GPi	5.2±1.2	5.9±0.9	0.2	8.3±2.5	8.9±3.8	0.6	STR-GPe	0.13±0.09	0.12±0.07	0.6
GPe	5.3±1.0	6.1±2.2	0.3	8.9±1.4	8.2±3.4	0.5	GPi-GPe	0.09±0.045	0.09±0.038	0.9

Supp. Table 2: Subgroup-Analysis of neurophysiological features between patients with cervical or segmental/generalized dystonia. Shown are mean ± standard deviation that are compared with permutation tests and FDR-corrected for multiple comparisons. Abbreviations: BG nucleus: Basal ganglia nucleus; GPi: Globus pallidus internus; GPe: Globus pallidus externus; LF: Low frequency (3-12 Hz); iCOH: imaginary part of coherence; Cervical: Cervical dystonia; Seg/Gen: Segmental or generalized dystonia; P-Val: p-Value for comparison between cervical and segmental/generalized dystonia group.

Parameter	T1w 3D MP-RAGE	T2-TSE	T1w 3D FGATIR
Repetition time, ms	2,300	13,320	3,000
Echo time, ms	2.32	101	3.44
Inversion time, ms	900	n.a.	414
Inversion pulse angle	90°	n.a.	180°
Field of view, mm	240 × 240	250 × 250	240 × 240
Slices, mm	192 × 0.9	70 × 2.0	160 × 1
Orientation	Sagittal	Axial	Axial
Bandwidth, Hz/Px	200	217	130
Acquisition time, min	5:21 min	4:15 min	6:17 min
Voxel size, mm^3	0.9 × 0.9 × 0.9	0.7 × 0.7 × 2.0	0.9 × 0.9 × 1.0

Supplementary table 3. Imaging Sequences and Acquisition Parameters Employed During Preoperative MRI. Note that sequences were obtained on a 3.0T clinical MRI scanner (Skyra or Vida Magnetom, Siemens, Erlangen, Germany), and parameters were optimized accordingly. FGATIR can be implemented on 1.5T scanners; however, parameters would have to be adjusted accordingly. 3D = 3 dimensional; FGATIR = fast gray matter acquisition T1 inversion recovery; MP-RAGE = magnetization-prepared rapid acquisition gradient echo; n.a. = not applicable; Px = pixel; T1w = T1-weighted; TSE = turbo spin echo.
